# Australian Podiatry Conference 2025

**DOI:** 10.1002/jfa2.70116

**Published:** 2026-02-02

**Authors:** Peta Tehan, Andrew Buldt, Alicia James

**Affiliations:** ^1^ School of Clinical Sciences Monash University Melbourne Australia; ^2^ Discipline of Podiatry, La Trobe Sport and Exercise Medicine Research Centre School of Allied Health, Human Services and Sport La Trobe University Melbourne Australia; ^3^ Allied Health Department Monash Health Melbourne Australia

Here, we present abstracts from the Australian Podiatry Conference 2025, which had the theme ‘Unleashing potential’. The conference was held from 26 to 28 June at the Gold Coast Convention and Exhibition Centre. This was the largest conference to date, with 1070 delegates in attendance. A total of 117 abstracts were presented by 109 speakers and are published in the pages that follow. This showcases the current activity and diversity of local podiatry research.

Several different themes were derived based on submissions, some of which included high‐risk foot care, paediatrics, First Nations health, musculoskeletal and sports, and aged care. This is reflective of the key clinical areas that podiatrists work within. There was also strong representation from technology and digital innovation. Sessions on artificial intelligence and new technologies demonstrated that podiatrists are adopting technological change and are interested in its potential. Some of the included abstracts published here represent work in progress: preliminary findings, pilot studies, quality improvement initiatives and innovative clinical approaches. Some report fully developed research programmes, whereas others present emerging ideas or practice innovations. We encourage readers to engage critically with this work. As you review these abstracts, we hope you find inspiration for your own practice and research and gain fresh perspectives on the challenges and opportunities before us. In line with our theme, these abstracts demonstrate the potential in Australian podiatry research.

## Conflicts of Interest

The authors declare no conflicts of interest.

## Data Availability

The authors have nothing to report.

